# PET/CT imaging of head-and-neck and pancreatic cancer in humans by targeting the “Cancer Integrin” αvβ6 with Ga-68-Trivehexin

**DOI:** 10.1007/s00259-021-05559-x

**Published:** 2021-09-24

**Authors:** Neil Gerard Quigley, Katja Steiger, Sebastian Hoberück, Norbert Czech, Maximilian Alexander Zierke, Susanne Kossatz, Marc Pretze, Frauke Richter, Wilko Weichert, Christian Pox, Jörg Kotzerke, Johannes Notni

**Affiliations:** 1 Institute of Pathology, Technische Universität München, Munich, Germany; 2 Klinik und Poliklinik für Nuklearmedizin, Universitätsklinikum Carl Gustav Carus an der Technischen Universität Dresden, Dresden, Germany; 3 Center of Nuclear Medicine and PET/CT Bremen, Bremen, Germany; 4 Department of Nuclear Medicine, University Hospital Klinikum rechts der Isar, and Central Institute for Translational Cancer Research (TranslaTUM), Technical University of Munich, Munich, Germany; 5 Clinic of Internal Medicine, Hospital St. Joseph-Stift Bremen, Bremen, Germany; 6 Experimental Radiopharmacy, Clinic for Nuclear Medicine, University Hospital Essen, Hufelandstr. 55, 45147 Essen, Germany

**Keywords:** Positron emission tomography, Carcinoma, Integrins, Gallium-68

## Abstract

**Purpose:**

To develop a new probe for the αvβ6-integrin and assess its potential for PET imaging of carcinomas.

**Methods:**

Ga-68-Trivehexin was synthesized by trimerization of the optimized αvβ6-integrin selective cyclic nonapeptide Tyr2 (sequence: c[YRGDLAYp(*N*Me)K]) on the TRAP chelator core, followed by automated labeling with Ga-68. The tracer was characterized by ELISA for activities towards integrin subtypes αvβ6, αvβ8, αvβ3, and α5β1, as well as by cell binding assays on H2009 (αvβ6-positive) and MDA-MB-231 (αvβ6-negative) cells. SCID-mice bearing subcutaneous xenografts of the same cell lines were used for dynamic (90 min) and static (75 min p.i.) µPET imaging, as well as for biodistribution (90 min p.i.). Structure–activity-relationships were established by comparison with the predecessor compound Ga-68-TRAP(AvB6)_3_. Ga-68-Trivehexin was tested for in-human PET/CT imaging of HNSCC, parotideal adenocarcinoma, and metastatic PDAC.

**Results:**

Ga-68-Trivehexin showed a high αvβ6-integrin affinity (IC_50_ = 0.047 nM), selectivity over other subtypes (IC_50_-based factors: αvβ8, 131; αvβ3, 57; α5β1, 468), blockable uptake in H2009 cells, and negligible uptake in MDA-MB-231 cells. Biodistribution and preclinical PET imaging confirmed a high target-specific uptake in tumor and a low non-specific uptake in other organs and tissues except the excretory organs (kidneys and urinary bladder). Preclinical PET corresponded well to in-human results, showing high and persistent uptake in metastatic PDAC and HNSCC (SUV_max_ = 10–13) as well as in kidneys/urine. Ga-68-Trivehexin enabled PET/CT imaging of small PDAC metastases and showed high uptake in HNSCC but not in tumor-associated inflammation.

**Conclusions:**

Ga-68-Trivehexin is a valuable probe for imaging of αvβ6-integrin expression in human cancers.

**Supplementary Information:**

The online version contains supplementary material available at 10.1007/s00259-021-05559-x.

## Introduction

Cancer possesses a uniquely terrifying quality in the perception of the general public—barely understood, frequently being personificated, used as a metaphor, or even mistaken as a personal trait [1]. Although other devastating pandemics have occasionally taken the lead in public agitation and fear (e.g., AIDS and recently COVID-19), cancer may still be seen as “The Emperor of All Maladies” [2], for its incidence rate in a globally ageing population that naturally grows. Already Galen reserved the metaphorical term “cancer” to the malignant and deadly forms of solid neoplasia. Malignancy, in turn, is tantamount to infiltrative growth, metastasis, and dedifferentiation since Virchow’s time [3]. Among the molecular factors governing malignancy, integrins, a class of 24 transmembrane cell adhesion receptors which are each formed by dimerization from 18 α- and 8 β-subunits, are not always the first that come to mind. Although awareness is mostly limited to the subtype αvβ3 because of its well-established role as a promoter of (tumoral) angiogenesis [4, 5], many other integrins are involved in every step of cancer development [6]. The dimer αvβ6, which is solely expressed by epithelial cells, is arguably most intertwined with carcinogenesis [7]. It drives at least two acquired capabilities of tumor cells as defined by the hallmarks of cancer [8], notably, insensitivity to anti-growth signals, and, additionally, invasiveness and metastasis. The signature functionality of αvβ6-integrin is the activation of transforming growth factors beta 1 and 3 (TGF-β’s) [9], which are secreted by virtually all mammalian cell types into the extracellular space in the form of an inactive complex with latency-associated peptide (LAP) [10]. αvβ6-integrin binds to an Arg-Gly-Asp (RGD) sequence of LAP and, by exerting a pulling force along the longitudinal axis of the β6 protein [11], distorts the conformation of LAP and thereby ultimately releases TGF-β [12]. The link to tumor invasion is given by the fact that tumor cells frequently develop a resistance against the overall growth-inhibiting effect of TGF-β [13] by a loss of certain downstream signaling components, e.g., p53 [14] or Smad4 [15]. Such tumor cells can benefit from generating a high TGF-β concentration in their vicinity, because it suppresses only the growth of normal cells and consequently facilitates infiltration [16]. In short, the epithelium-only αvβ6-integrin initiates and promotes the infiltrative growth of many kinds of malignant epithelial neoplasia—nowadays synonymous to “carcinoma,” a term that was originally coined about 400 BC by Hippocrates (“karkinos” is Greek for crayfish) and ≈ 400 years later latinized by Celsus to “cancer.” Of all integrins, expression of αvβ6 is apparently most tightly connected to what is known ever since as the archetype of cancer, which is why we chose to refer to it as the “cancer integrin” [17].

Consistent with its role of promoting invasion, the highest αvβ6-integrin expression density is often found at the boundary of tumor- and healthy tissue [18]. There, it also drives tissue remodeling by epithelial-mesenchymal transition (EMT) via the TGF-β pathway and the interaction with tumor stroma. Cells of stroma-rich neoplasia such as pancreatic ductal adenocarcinoma (PDAC) thus frequently show concomitant αvβ6-integrin expression [19, 20]. We found a high expression of the β6 subunit on tumor cells in 88% of nearly 400 specimen of pancreatic ductal adenocarcinoma (PDAC) primaries, in 14 out of 15 metastases, and in 57% of grade 3 pancreatic intraepithelial neoplasia (PanIN3; immediate PDAC precursor lesions) [21]. Its prevalence in PDAC but also in other carcinomas, most importantly, squamous cell [18], basal cell, lung adeno, and colon [22] makes it a highly attractive target for tumor imaging [23]. αvβ6-integrin is furthermore expressed in the course of other conditions associated with TGF-β-driven extracellular matrix (ECM) remodeling such as idiopathic pulmonary fibrosis (IPF) [24], thereby further expanding the potential field of applications.

**Fig. 1:**
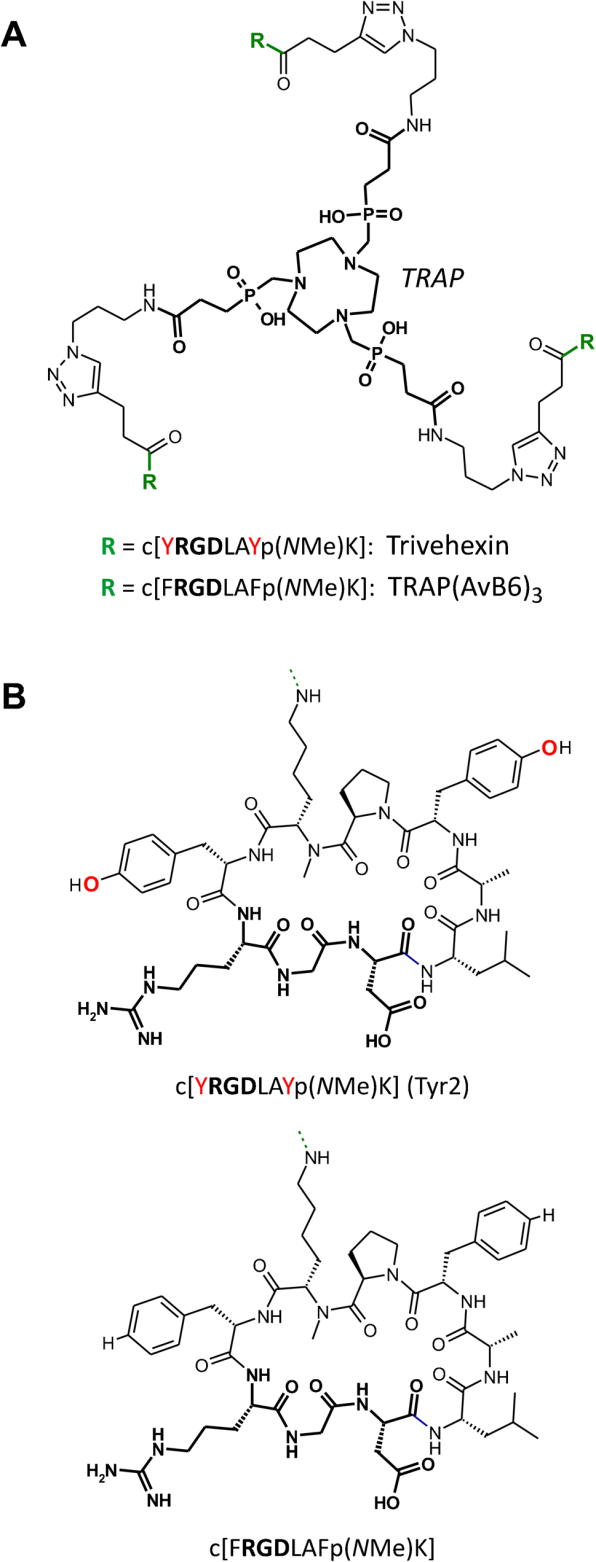
Chemical structures of peptides and conjugates. **A** Structure of the trimers TRAP(AvB6)_3_ and Trivehexin, which were synthesized by “click-chemistry” (i.e., copper(I) catalyzed azide-alkyne cycloaddition, CuAAC) conjugation of the TRAP chelator core (highlighted in boldface). **B** Structures of the peptides used, with their RGD sequences highlighted in boldface. On an atomic level, the structural differences merely consists of two additional oxygens of Tyr2 (sequence: c[YRGDLAYp(*N*Me)K]) (colored in red)

A variety of αvβ6-integrin targeted PET radiopharmaceuticals have therefore been developed in recent years. Some were already tested in humans [25–29] or even employed in clinical trials [30–32], thus underscoring the clinical potential of αvβ6-integrin imaging in human carcinomas and in IPF. Non-specific uptakes in stomach or intestines, and to a lesser extent, also in liver, lung or pancreas, might however challenge the respective diagnostic procedures. Such occasionally observed uptakes are prone to compromising the unambiguous identification of tumors and particularly of metastases or raise the detection limit for low-level target expression. Along these lines, we earlier reported ^68^ ﻿Ga-Avebehexin [17], a ^68^ Ga-labeled monomeric triazacyclononane-triphosphinate (TRAP) [33] conjugate of αvβ6-integrin-specific cyclic nonapeptide c[FRGDLAFp(*N*Me)K] [34], as well as a series of other ^68^ Ga- and ^177^Lu-labeled conjugates thereof [35]. Most of these compounds showed a very low background and thus a good tumor-to-background contrast in rodent models, but their tumor accumulation was ultimately too low for a successful clinical transfer. Although we repeatedly observed that the trimeric ^68^ Ga-TRAP conjugates of integrin ligands showed a much higher affinity, improved selectivity, enhanced tumor uptake, and prolonged retention [36–40], this strategy did not work out for c[FRGDLAFp(*N*Me)K]. A ^68^ Ga-labeled TRAP trimer thereof, referred to as ^68^ Ga-TRAP(AvB6)_3_ (Fig. 1), showed a very unfavorable biodistribution [17] and required further optimization. We now report the successful application of the novel, improved ^68^ Ga-labeled trimerized αvβ6-integrin selective nonapeptide ^68^ Ga-Trivehexin (Fig. 1) for imaging of human carcinomas, thus transforming the concept of multivalent molecular imaging probes from a future vision [41] into clinical reality.

## Materials and methods

### General

Materials, reagents, general procedures, and instrumentation used for chemical syntheses and analyses have been detailed elsewhere [39]. In addition to what has been reported before, the building block TRAP(azide)_3_ was purchased from *Fosfinos s.r.o.* (Kladno, CZ). c[FGDLAFp(*N*Me)K(pentynoic amide)] and TRAP(AvB6)_3_ were synthesized according to the literature [17]. Previously described protocols were followed for determination of integrin affinities by ELISA on immobilized integrins [42] and for β6-integrin immunohistochemistry (IHC) [17].

### Compounds

A detailed description of the synthesis of the building block Tyr2-alkyne, c[YRGDLAYp(*N*Me)K(pentynoic amide)], and the labeling precursor Trivehexin is provided in the Supporting Information.

#### Radiolabeling

Radiolabeling for preclinical experiments and determination of *n*-octanol–PBS distribution coefficients (log *D*_7.4_) was done as described [39]. Applying this protocol, 5 nmol (0.5 nmol for cellular assays in order to maximize molar activity) of TRAP(AvB6)_3_ or Trivehexin were labeled with ^68^ Ga at pH 2, resulting in RCY > 95%. The purity of the radiolabeled compounds was confirmed by radio-TLC, using silica impregnated glass fiber chromatography paper (ITLC® by *Agilent*) as stationary phase and 0.1 M aq. sodium citrate as mobile phase (purities were > 99%).

Radiolabeling for clinical testing was performed by an automated procedure on a Modular-Lab module (Eckert & Ziegler, Berlin) connected to a ^68^Ge/^68^ Ga-Generator (GalliaPharm® by *Eckert & Ziegler*, Berlin, Germany) in a sterile hot cell as described earlier [40]. The final product (450 ± 45 MBq, starting from ~ 600 MBq) was diluted with 0.15 M phosphate-buffered saline (9 mL; SC-01-PbS, ABX, Radeberg, Germany). The QC release criteria (endotoxin level < 5.00 EU/mL; radio-TLC > 99%; radio-HPLC > 95%; pH 4.0–8.0) were met for all radiosyntheses. Sterility was tested retrospectively after complete decay (3 d post synthesis).

### Cellular uptake assay

H2009 human lung adenocarcinoma cells (CRL-5911) and MDA-MB-213 cells (HTB-26; both from *American Type Culture Collection (ATCC)*, Manassas, VA, USA) were cultivated as recommended by the distributor. Cells were grown in a monolayer culture at 37 °C in a 5% CO_2_ humidified atmosphere. All cells were tested regularly to exclude mycoplasma contamination.

αvβ6-dependent uptake of ^68^ Ga-Trivehexin was determined in H2009 (αvβ6-positive) and MDA-MB-231 (αvβ6-negative) cells. Ten thousand cells per well were seeded into a 96-well plate and incubated with a 0.3 nM solution of ^68^ Ga-Trivehexin (30 kBq/well; molar activity: 452–558 MBq/nmol) for 1 h at 37 °C. For blocking, ^nat^Ga-Trivehexin was added prior to the radioligand at a final concentration of 250 nM. At the end of the incubation time, cells were pelleted and washed twice, followed by cell lysis with a 1-M NaOH solution. Supernatants and cell lysates were collected and measured on a Wizard^2^ automated gamma counter (PerkinElmer). The cellular uptake of ^68^ Ga-Trivehexin was quantified as percent of total radioactivity added. This experiment was repeated 6 × (H2009) or 4 × (MDA-MB-213) with 5 or 3 technical replicates, respectively.

### Animal experiments

All animal experiments were approved by the responsible authority (Regierung von Oberbayern) and have been performed in accordance with general animal welfare regulations in Germany and the institutional guidelines for the care and use of animals. Keeping of the animals, generation of subcutaneous H2009 and MDA-MB-231 tumor xenografts, ex-vivo biodistribution studies, and µPET imaging were done according to previously described protocols [39]. Multiplicity of PET and biodistribution studies ranged from 3 to 5; animal numbers for each individual experiment are denoted in the respective data tables in the Supporting Information and, additionally, in the Results section. The metabolic stability of ^68^ Ga-Trivehexin has been determined in one mouse, according to a previously published protocol [36].

### Patients

The synthesis and quality control of ^68^ Ga-Trivehexin for clinical application was done applying a previously described protocol [40], using 10 nmol of Trivehexin. Application of ^68^ Ga-Trivehexin in human was done according to §13/2b of the German Drug Act (Arzneimittelgesetz). All human participants provided written informed consent, also for publication of images, prior to the investigation. Following iv administration of ^68^ Ga-Trivehexin, there were no adverse or clinically detectable pharmacologic effects. No significant changes in vital signs or the results of laboratory studies or electrocardiograms were observed. Data for a total of 4 human subjects were included in this report. The PET/CT for patient #1 was performed according to a previously published protocol, as was calculation of the dosimetric parameters [40].

Patient #2 (Fig. 4A + B) underwent both [^18^F]FDG and ^68^ Ga-Trivehexin PET/CT within 2 weeks for primary staging of a recently detected, locally advanced p-16 negative head-and-neck squamous cell carcinoma (HNSCC) of the oropharynx with infiltration of the lips and ipsilateral cervical lymph node metastases (cT4a cN2b cM0). A panendoscopy and tracheostomy were carried out 4 weeks prior to the [^18^F]FDG. The tumor was not suitable for surgical removal and a simultaneous radiochemotherapy was indicated.

Patient #3 (Fig. 4C–E) was diagnosed with an adenocarcinoma (salivary duct carcinoma) in the right parotitis in 2019 and initially underwent total parotidectomy and unilateral neck dissection (pT2 pN1b [21/23] L1 V1 Pn1 R1). A post-resection and contralateral neck-dissection was performed 1 month later, followed by adjuvant radiation. In 2020 the patient developed prelaryngeal and lateral orbita angle metastases, which were resected and adjuvantly irradiated. A follow-up MRI revealed a new subcutaneous prelaryngeal metastasis. Consecutively the patient underwent [^18^F]FDG and ^68^ Ga-Trivehexin PET/CT within 2 days. Since no further tumor manifestations were present in both examinations, surgical resection was indicated and performed. β6-integrin IHC [21] was done for specimen of the excised metastasis.

Patient #4 was diagnosed with a carcinoma of the pancreatic head by endosonography and CT morphology. Pancreatic adenocarcinoma was verified by biopsy. A PET/CT scan using ^68^ Ga-Trivehexin was performed 120 min after i.v. administration, showing a high tracer uptake in the pancreatic tumor burden and multiple liver metastases. According to this finding, surgical treatment was rejected and a chemotherapy regimen was started.

## Results

### Synthesis and in vitro characterization

Similar to TRAP(AvB6)_3_ [17] or the previously reported αvβ8-integrin tracer ^68^ Ga-Triveoctin [40], the trimeric conjugate Trivehexin was synthesized by conjugation of the Lys(pentynoic amide) derivative of Tyr2 to the symmetrical TRAP chelator scaffold [43] (TRAP = 1,4,7-triazacyclononane-1,4,7-tris[methylene(2-carboxyethyl)]phosphinic acid) [44], applying a convenient and straightforward click chemistry (CuAAC) protocol [45] (Fig. 1). Automated ^68^ Ga labeling of 5 nmol of Trivehexin for preclinical characterization afforded ^68^ Ga-Trivehexin with radiochemical yields exceeding 95% and purity (radio-TLC) of > 99%.

The exchange of both phenylalanines in c[FRGDLAFp(*N*Me)K] to tyrosines was done with the intention to increase the hydrophilicity of the resulting conjugates, because we observed earlier that TRAP- c[FRGDLAFp(*N*Me)K] trimers possessed two-digit picomolar αvβ6-integrin affinities but were essentially unusable because of the concomitant increase of non-specific organ uptakes [17]. Replacement of each individual Phe in c[FRGDLAFp(*N*Me)K] (*IC*_50_ = 0.26 pM) [34] by Tyr was shown earlier to result in only slightly lower affinities (*IC*_50_ of 0.39 and 0.40 pM for c[FRGDLAYp(*N*Me)K] and c[YRGDLAFp(*N*Me)K]), respectively) [34]. Simultaneous Phe-to-Tyr exchange at both positions leads to a notably lower *IC*_50_ and reduced subtype selectivities of the building block Tyr2-alkyne, as compared with its phenylalanine congener (see Table 1). This drawback was however compensated by trimerization, which, in accordance with previous observations [39], resulted in a substantially higher αvβ6-integrin affinity (18-fold) and a substantial attenuation of some selectivity factors (Table 1). Finally, ^68^ Ga-Trivehexin also showed the desired increase in hydrophilicity, its *n*-octanol-PBS distribution coefficient (log *D*_7.4_ =  − 2.1 ± 0.1) being moderately lower than that of ^68^ Ga-TRAP(AvB6)_3_ (re-determined, log *D*_7.4_ =  − 1.5 ± 0.1).

**Table 1:** Integrin affinities, expressed as 50% inhibition constants (*IC*_50_), and *IC*_50_-based selectivities

Compound	*IC*_50_ (95% confidence interval) [nM]				αvβ6-selectivity over		
	αvβ6	αvβ8	αvβ3	α5β1	αvβ8	αvβ3	α5β1
c[FRGDLAFp(*N*Me)K(pentynoic amide)]	0.17 (0.09–0.33)	32 (20–51)	424 (270–670)	226 (115–193)	188	2494	1329
c[YRGDLAYp(*N*Me)K(pentynoic amide)] (Tyr2-alkyne)	0.84 (0.56–1.2)	26 (19–37)	219 (88–540)	150 (116–193)	31	261	179
^nat^Ga-Trivehexin	0.047 (0.030–0.074)	6.2 (2.8–14)	2.7 (1.2–6.0)	22 (16–30)	131	57	468

### Preclinical evaluation

**Fig. 2:**
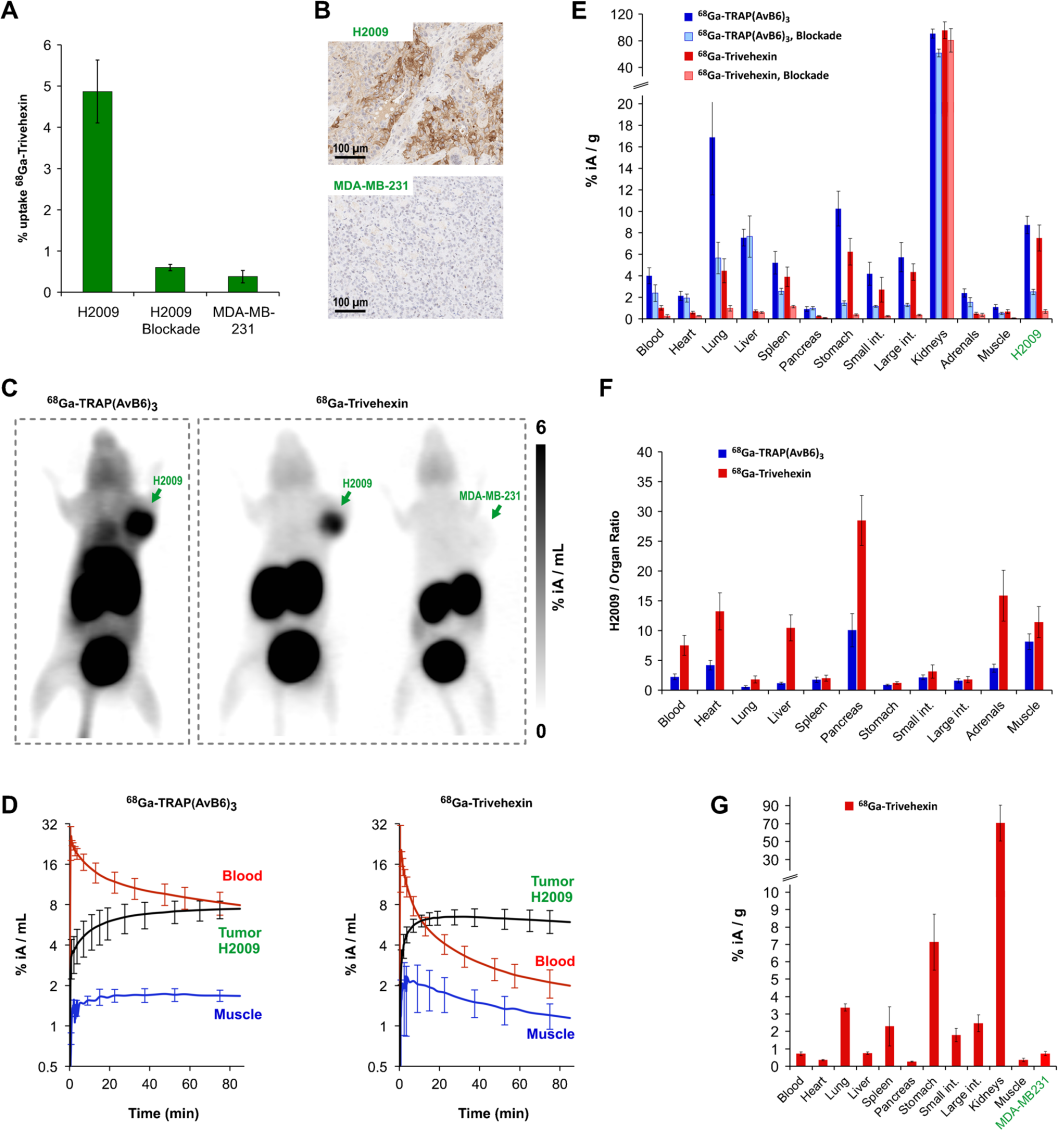
Preclinical development of ^68^ Ga-Trivehexin. Data were generated using H2009 (αvβ6^+^ human lung adenocarcinoma) or MDA-MB-231 (αvβ6^−^ human triple-negative breast cancer) cell lines and murine xenografts thereof. **A** Cellular uptake of ^68^ Ga-Trivehexin with and without blocking with ^nat^Ga-Trivehexin. Mean and SD of n = 4–6 biological repeats. **B** β6-integrin immunohistochemistry (IHC) confirms moderate αvβ6-integrin expression in H2009 and absence of αvβ6 in MDA-MB-231 tumor xenografts. **C** Static µPET images (maximum intensity projections), 75 min p.i., recording time 20 min. The same H2009-tumor bearing animal was used for both PET scans shown, with a 24-h recovery period. **D** Biokinetics of ^68^ Ga-labeled trimers, derived from dynamic PET scans (averages ± SD for 3 independent scans). **E** Biodistribution of trimers in H2009-bearing animals. Dark bars indicate controls (injected molar amounts of approx. 100 pmol, *n* = 5); light bars of the same color show blockade (50 nmol unlabeled, administered 10 min before the radioactive compound, *n* = 3). **F** Tumor-to-organ ratios derived from H2009 biodistribution data. **G** Biodistribution of ^68^ Ga-Trivehexin (105 ± 34 pmol) in MDA-MB-231-bearing animals (*n* = 5). – Numerical data for graphs shown in **E**, **F**, and **G**, including the exact values of injected amounts, are provided in the Supporting Information, Tables S1–3

The αvβ6-integrin expressing human lung adenocarcinoma cell line H2009 and the αvβ6-negative breast cancer cell line MDA-MB-231 were used for cellular assays and in-vitro experiments. ^68^ Ga-Trivehexin showed specific (i.e., blockable) uptake in H2009 but not in MDA-MB-231 cells (Fig. 2A). Both ^68^ Ga-labeled trimers were further evaluated in severe combined immunodeficient (SCID) mice, bearing subcutaneous xenografts of the same cell lines on the right shoulders. Dynamic µPET revealed that ^68^ Ga-Trivehexin was cleared much faster from the blood than ^68^ Ga-TRAP(AvB6)_3_ (Fig. 2D). In addition, it showed virtually no unfavorable non-specific accumulation (i.e., non-blockable uptake) in many organs, which was observed for ^68^ Ga-TRAP(AvB6)_3_ particularly in the liver and the lung, but also in the tumor tissue (Fig. 2E). A determination of metabolites in blood and tissues according to our standard protocol [36] unexpectedly failed because no activity could be extracted after workup, presumably owing to very strong protein binding. However, HPLC analysis of the urine showed no signs of metabolic degradation (see Supporting Information, Figure S6).

Of note, there were still blockable ^68^ Ga-Trivehexin uptakes in, e.g., lung and stomach, which are related to proven physiological β6-integrin expression in mice [17]. The improved biodistribution profile translated to substantially enhanced tumor-to-organ ratios for ^68^ Ga-Trivehexin (Fig. 2F) and furthermore to µPET images with lower background and improved tumor delineation (Fig. 2C). Specificity for αvβ6-expressing tumors was confirmed by PET and biodistribution in MDA-MB-231 xenografts. β6-IHC confirmed that in contrast to H2009 tumors, MDA-MB-231 xenografts were β6-negative (Fig. 2B), which is why virtually no ^68^ Ga-Trivehexin uptake was observed in PET and biodistribution (Fig. 2C + G).

### In-human dosimetry estimate

**Fig. 3:**
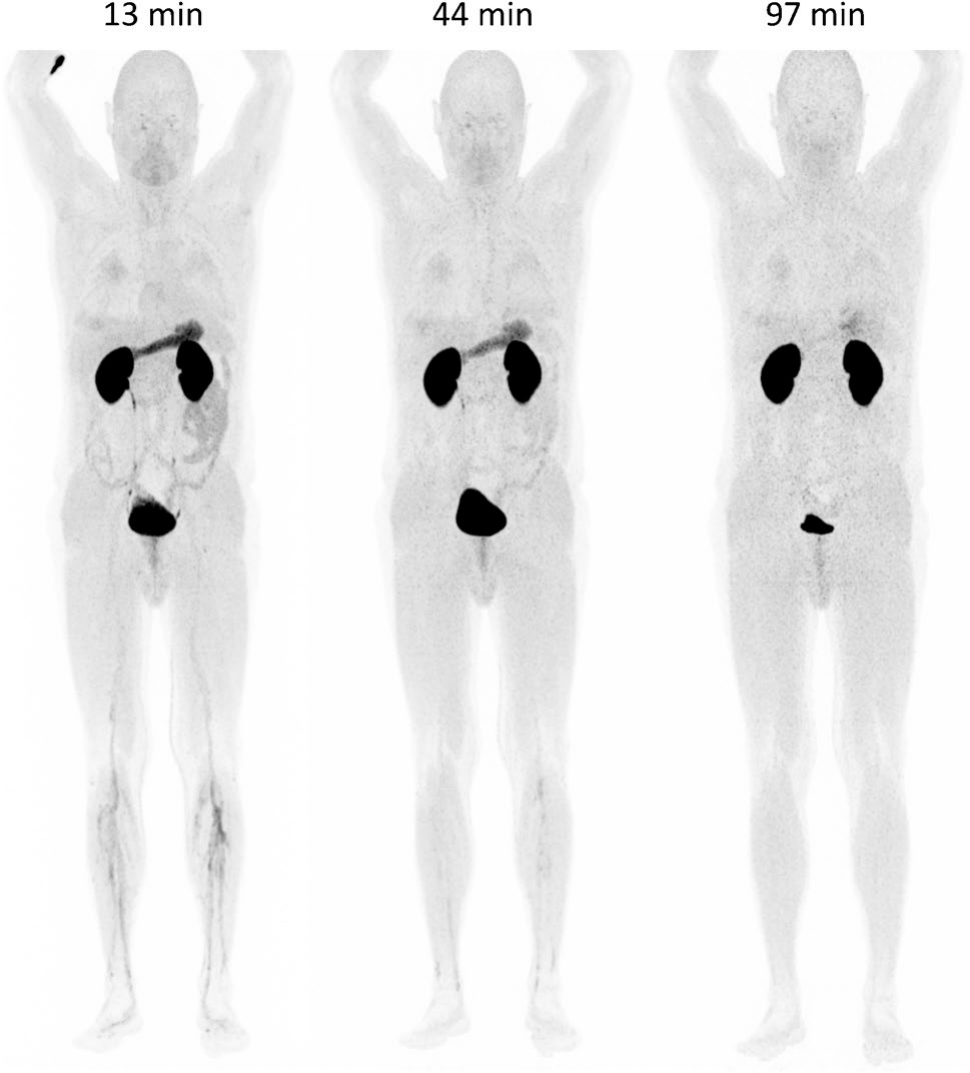
^68^ Ga-Trivehexin PET of patient #1, 172 MBq, total peptide amount: 5 nmol, start times p.i. denoted above anterior maximum intensity projections (MIPs) scaled to SUV 15. Apart from kidney and urinary bladder, a significant uptake is noted in the (empty) stomach, which however had disappeared 1.5 h after injection. PET at 1 h p.i. or later is thus preferred. For organ SUVs and dosimetry data, see Supporting Information, Tables S4 and S5

Whole-body PET images (Fig. 3) showed a biodistribution generally characterized by a low soft tissue uptake. Apart from the renal excretion and partial retention, relevant uptakes were registered only in the gastric mucosa and the small intestine (for SUV values see Supporting Information Table S4). Currently, we are not able to provide a rationale for the transient uptake in the upper gastrointestinal tract, which decreased analogously to the vascular tracer retention rapidly over time and was nearly absent in the late image. We thus found that ^68^ Ga-Trivehexin PET should ideally be performed at time points > 60 min p.i.. With a biological half-life of 10 h (renal excretion) and an assumed urinary bladder residence of 0.07 h, dosimetry calculations using OLINDA V1.1 yielded a moderate effective dose (ICRP 60) of 3.36E-02 mSv/MBq, or 4.7 mSv for an injection of 148 MBq (4 mCi) ^68^ Ga-Trivehexin (for individual organ dosimetry factors see Supporting Information Table S5).

### Imaging of carcinomas in human

The tumor configuration and tracer uptake of both a locally advanced oropharyngeal HNSCC (Fig. 3A + B) and a prelaryngeal cutaneous metastasis of an initially resected right sided parotideal adenocarcinoma (Fig. 3C + D) appear quite similar in the ^68^ Ga-Trivehexin- and [^18^F]FDG-PET. In the HNSCC patient #2, the inflammatory, peristomal uptake around the tracheostoma was lower in the ^68^ Ga-Trivehexin study as compared with the [^18^F]FDG-PET done 2 weeks before. Reactively increased lymphatic uptake in the pharyngeal tonsil as well as in periclavicular, mediastinal, and hilar lymph nodes occurred only in [^18^F]FDG imaging.

**Fig. 4:**
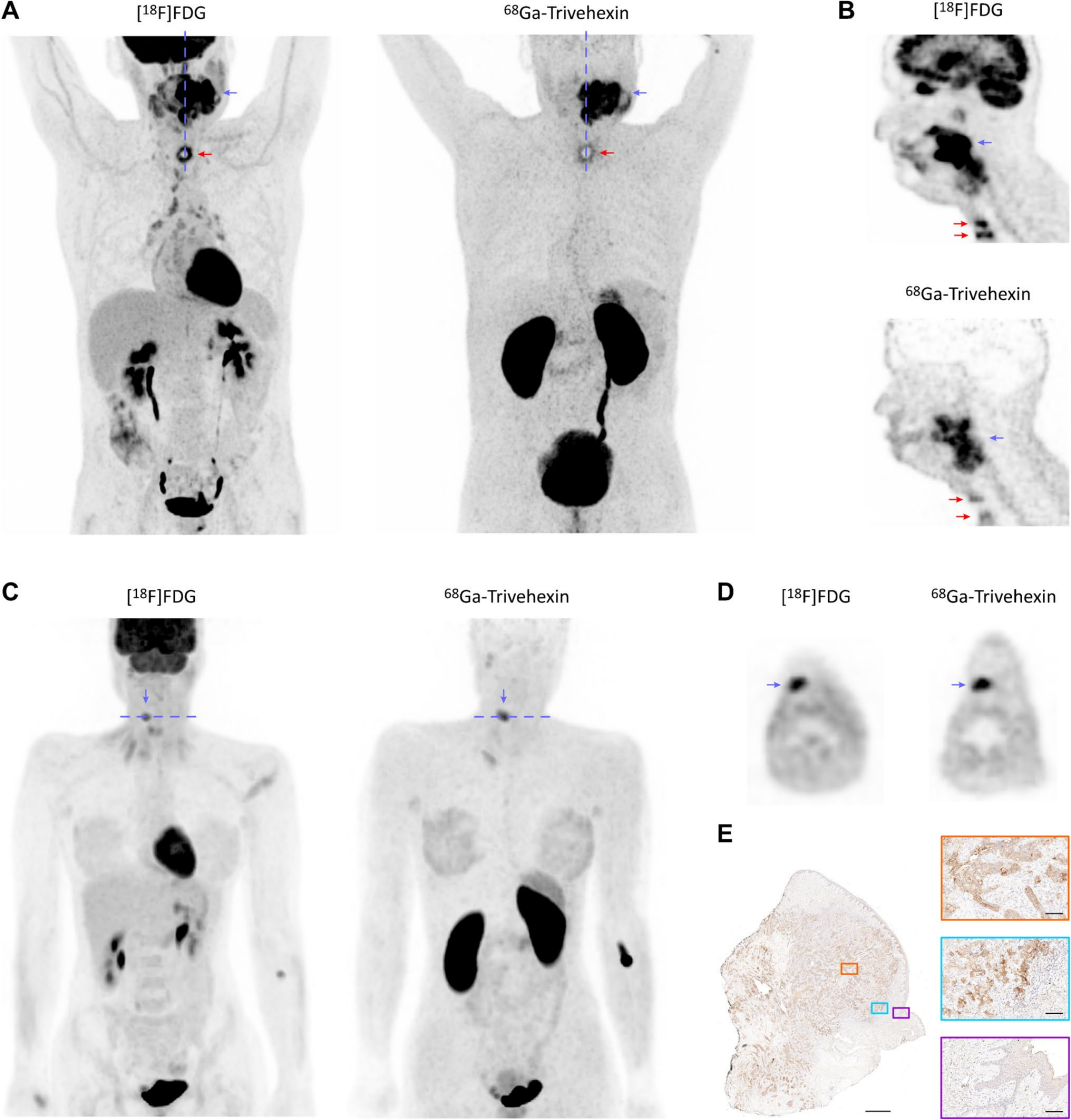
[^18^F]FDG and corresponding ^68^ Ga-Trivehexin PET of head-and-neck cancers (locations indicated by blue arrows, slice planes by blue dashed lines). All PET images are scaled to SUV 8, except **D** (to SUV 5). **A** PET (MIPs) of HNSCC patient #2 (m, 61 y, 70 kg; 331 MBq [^18^F]FDG 62 min p.i.; 142 MBq ^68^ Ga-Trivehexin, *A*_M_ = 27 MBq/nmol, total peptide amount 5.3 nmol, 61 min p.i.) with locally advanced oropharyngeal carcinoma and several adjacent unilateral lymph node metastases. ^68^ Ga-Trivehexin showed uptake in the primary and metastases (SUV_max_ = 13.9 and 12.9, respectively) and in a lesser extent around a 7-week-old tracheostoma (red arrows, SUV_max_ = 5.8; cf. 10.2 for [^18^F]FDG). **B** Sagittal slices corresponding to dashed lines in **A**. **C** PET (MIPs) of patient #3 (f, 37 y, 61 kg; 286 MBq [^18^F]FDG 71 min p.i.; 135 MBq ^68^ Ga-Trivehexin, *A*_M_ = 29 MBq/nmol, total peptide amount 4.7 nmol, 67 min p.i.) suffering from a solitary prelaryngeal cutaneous metastasis of a previously resected and irradiated left-sided parotideal adenocarcinoma. **D** Axial slices corresponding to dashed lines in **C**. SUV_max_ of the metastasis was 6.1 for [^18^F]FDG and 7.6 for ^68^ Ga-Trivehexin, metabolic tumor volume was 2.2 cm^3^. **E** β6-integrin IHC of excised tissue of the patient shown in **C** + **D**; magnifications are shown for the central tumor area (orange box), the infiltrative margin (cyan box), and non-tumor tissue (violet box). Bars represent 1 mm for overview and 100 µm for the magnifications, respectively

The prelaryngeal cutaneous metastasis of the adenocarcinoma patient #3 (Fig. 4D) showed a higher uptake of ^68^ Ga-Trivehexin (SUV_max_ = 7.6) than of [^18^F]FDG (SUV_max_ = 6.1), while both PET modalities indicated a metabolic tumor volume of 2.2 cm^3^. Immunohistochemistry for β6-Integrin (Fig. 4E) revealed a moderate cytoplasmic to membraneous positivity in 10% of the tumor cells with an increased ratio of positive neoplastic cells (25%) towards the infiltrative margins of the metastasis. This is consistent with the observation that head-and-neck carcinomas express αvβ6-integrin particularly at the invasive edge where tumor cells are in direct contact with the tumor-associated stroma [18]. In addition, both tracers showed a comparably low bifocal uptake in the mastoid cells on the right side, representing an inflammatory process, and a modest physiological uptake in the breast parenchyma.

**Fig. 5:**
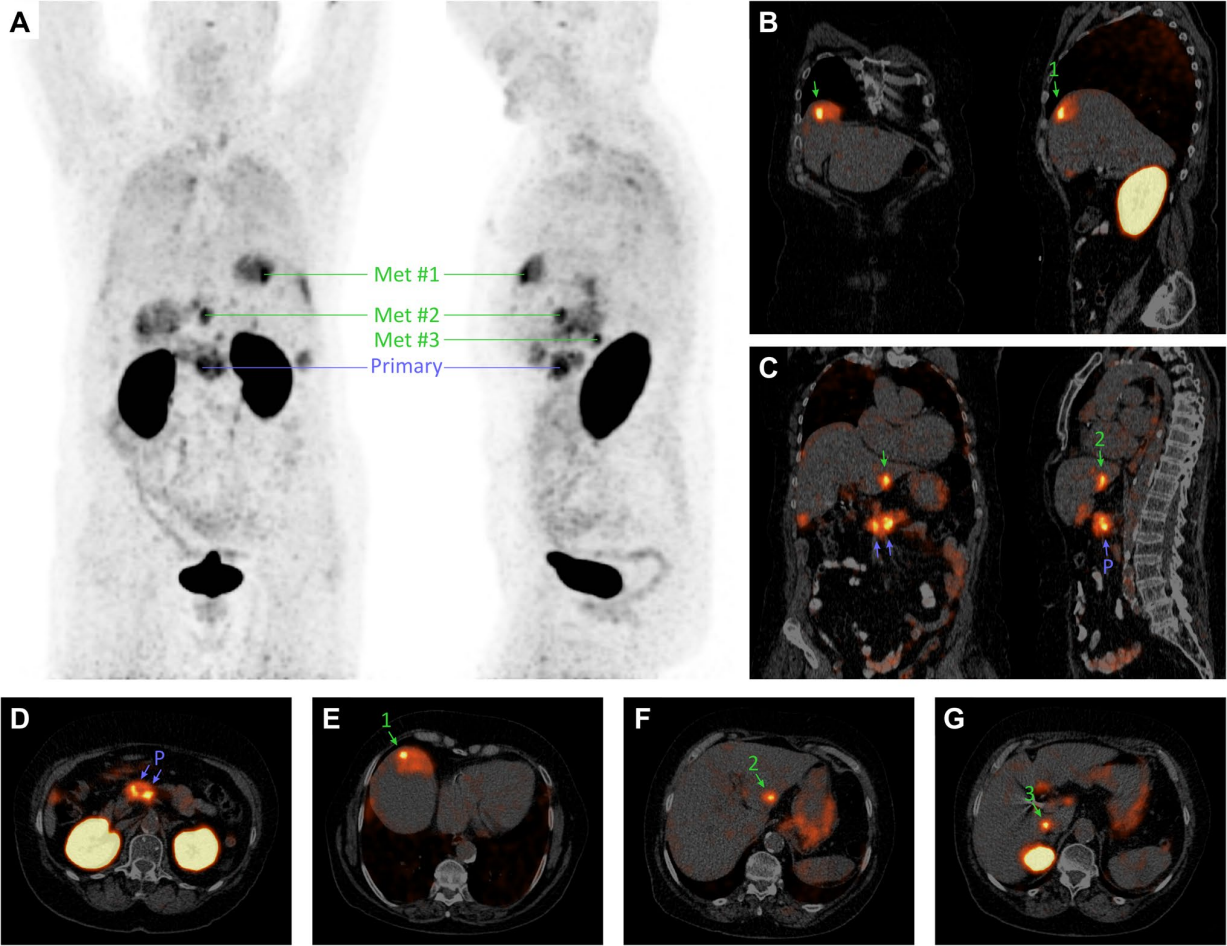
^68^ Ga-Trivehexin PET/CT of metastatic PDAC (confirmed by biopsy) in patient #4 (f, 80 y, 80 kg, 105 MBq, 120 min p.i.). PET is scaled to SUV 10 in all images. **A** Anterior and sagittal MIP, showing ^68^ Ga-Trivehexin uptake in the primary tumor (SUV_max_ = 9.8–10.9) and in 7 metastases. Selected metastases highlighted in panels **B**–**G** are indicated (SUV_max_ of #1, #2, and #3 are 10.9, 10.0, and 9.9, respectively). **B** Coronal and sagittal PET/CT slices through metastasis #1. **C** Coronal and sagittal PET/CT slices through primaries and metastasis #2. **D**, **E**, **F**, **G**, axial PET/CT slices though primaries and metastases #1, #2, and #3, respectively

The PET/CT shown in Fig. 5 is presenting two parts of a pancreatic tumor of patient #4, one placed in the region of pancreatic head passing over to the corpus and the other part more lateral passing from the corpus to the tail. The presence of tumor lesions was described by a CT scan only before, whereby one metastatic lesion was discovered in liver segment 8. Other liver lesions were interpreted as typical cysts by CT only but could be identified as PDAC metastases by ^68^ Ga-Trivehexin PET positivity. Of note, this scan was performed with a comparably low activity of 105 MBq and at a late time point of 120 min, i.e., after nearly two ^68^ Ga half-lives. Figure 5 thus demonstrates the capabilities of ^68^ Ga-Trivehexin PET, because clinically relevant information could be obtained from the images despite the low remaining total activity at scan time (approx. 30 MBq, less urinary excretion) which caused more background noise than in the PET images shown in Figs. 4 and 5.

## Discussion

### Strategy for optimization of biokinetics

In order to exploit the multimer effect to achieve a higher target affinity, enhanced uptake, and longer retention [41], we reasoned that increasing the intrinsic hydrophilicity of c[FRGDLAFp(*N*Me)K] should both accelerate blood clearance and reduce non-specific uptake of the corresponding trimers. An exchange of both phenylalanines by tyrosines, resulting in the cyclopeptide Tyr2 (see Fig. 1), appeared to be a convenient and simple strategy, yet at the risk of considerably lowering target affinity and selectivity which, fortunately, did not occur. αvβ6-integrin affinities of ^68^ Ga-TRAP(AvB6)_3_ [17] and ^68^ Ga-Trivehexin were in the same, low double-digit picomolar range (23 and 47 pM, respectively), and ^68^ Ga-Trivehexin showed a remarkable selectivity for αvβ6 over other RGD-binding integrins.

Of note, the difference between both cyclic peptide monomers is minimal on an atomic level. Tyr2 merely contains two additional oxygens (see Fig. 1), which means that the trimer ^68^ Ga-Trivehexin incorporates just 6 oxygen atoms more than ^68^ Ga-TRAP(AvB6)_3_, the gain in molecular weight amounting to only 96 Da. This is comparable to the mass of 2 polyethylene glycol (PEG) units ( (–CH_2_-CH_2_-O–)_2_, 88 Da), or approximately half of a monoglycosyl moiety ( C_6_H_12_O_6_, 180 Da), which has been attached as a polar modifier to RGD peptides before [46]. To our delight, ^68^ Ga-Trivehexin nonetheless exhibited dramatically improved biokinetics (see Fig. 2), above all, a rapid clearance from the blood pool, despite its rather modest increase in hydrophilicity (Δlog *D*_7.4_ = –0.6). With exception of the kidneys, non-specific uptakes were virtually completely eliminated, which is most appreciated for liver and lung because these organs are typical locations of primaries and metastases of αvβ6-integrin expressing carcinomas. The development of ^68^ Ga-Trivehexin showcases that it can be much more rewarding to carefully evaluate any possibility to optimize pharmacokinetics of radiopharmaceuticals by structural alteration of the respective bioligand itself, rather than attempting to mitigate unfavorable pharmacokinetics by PEGylation [17, 47] or other pharmacokinetic modifiers [46].

The comparison of ^68^ Ga-TRAP(AvB6)_3_ and ^68^ Ga-Trivehexin furthermore exemplifies that a successful multimerization might not in all cases be a strictly deterministic result of a thoughtful choice of scaffolds, linkers, symmetry, degeneracy coefficients, or other factors [41]. Multimers should rather be considered complex systems and their properties be perceived as emergent phenomena [48], leading to the simple truth that some receptor ligands are apparently suitable for multimerization whereas others, even those with an almost identical chemical structure and similar properties, are not [49]. Neither do we believe that the dramatically improved pharmacokinetics of ^68^ Ga-Trivehexin could have been predicted from any in vitro parameter, such as its affinity, selectivity, or its only moderately improved hydrophilicity. We therefore like to emphasize that the characteristics of more complex molecular systems, such as multimers, are to a considerable extent a consequence of their higher integrative level and thus, by principle, can neither be fully predicted on the basis of a few measured or calculated parameters nor concluded upon from the features of their constituents [48].

### Possible clinical implications of ^68^ Ga-Trivehexin PET

The preclinical and in-human PET results are largely in accordance. No relevant non-specific uptake was observed in any organ, except in the kidneys. Similar to the previously reported αvβ8-integrin tracer ^68^ Ga-Triveoctin, the kidney uptake observed in humans (SUV-based kidney-to-blood ratio of ≈ 29, 97 min p.i.) was lower than in mice (kidney-to-blood ratio of ≈ 93, 90 min p.i.). Overall, the presented preclinical and first-in-human data strongly suggest that ^68^ Ga-Trivehexin is indeed a suitable agent for non-invasive mapping of αvβ6-integrin expression in a clinical setting. The presented work is limited by the fact that only single cases have been examined, obviously not allowing for more than an educated guess on the future clinical impact of ^68^ Ga-Trivehexin PET. We nonetheless assume that the clinical scope encompasses all purposes that have been suggested for other αvβ6-integrin targeted radiopharmaceuticals before [17, 21, 25–32].

We particularly envisage an added value of ^68^ Ga-Trivehexin over [^18^F]FDG in cases where the latter shows a low sensitivity or specificity. Figure 4 demonstrates that ^68^ Ga-Trivehexin PET might allow for an improved delineation of head-and-neck cancers because it apparently shows a lower uptake in lymphatic tissue and tumor-associated inflammation. Such information might be valuable for planning external beam therapy regimen. In the same context, ^68^ Ga-Trivehexin PET might enable an improved follow-up because it could facilitate the assessment of an early response to radiation therapy, which sometimes is not easily possible with [^18^F]FDG due to its pronounced uptake in tissues afflicted by radiogenic inflammation. ^68^ Ga-Trivehexin might furthermore enable a more specific and sensitive diagnosis of metastatic carcinomas, particularly those which are characterized by a low metabolic conversion rate and/or a high fraction of stroma. Among those, pancreatic carcinoma is arguably the most important target, because it is one of the cancers with the worst overall prognosis and [^18^F]FDG possesses only a limited value for early detection of PDAC [50]. Figure 5 admittedly does not allow for more than a first glimpse on ^68^ Ga-Trivehexin-driven PET diagnostics of metastatic PDAC. We nevertheless hold the view that the demonstrated feasibility of detecting PDAC primaries and even small metastases might turn out to be a game changer, all the more because it also implicates the possibility of a differential diagnosis of PDAC vs. pancreatitis, or even the detection of αvβ6-integrin expressing PDAC precursor lesions, pancreatic intraepithelial neoplasias (PanIN) [21]. Although not supported by the currently available data, we furthermore imagine that ^68^ Ga-Trivehexin might turn out to be a suitable agent for monitoring the response to established PDAC chemotherapies like folfirinox and, as such, might claim a position in regular PDAC treatment regimen. Such applications, however, obviously need to be firmly established in future clinical trials. The same applies to other potential uses, e.g., imaging of fibrosis [29] or monitoring of the response to anti-fibrotic inhalation therapy with αvβ6-integrin antagonists [31, 32].

## Conclusion

Trimerization of the αvβ6-integrin binding cyclic nonapeptide Tyr2 on the TRAP chelator core resulted in the novel PET probe ^68^ Ga-Trivehexin, which showed an exceptional αvβ6-integrin affinity of 47 pM, a high selectivity over other RGD-binding integrin subtypes and enabled sensitive and specific imaging of αvβ6-integrin expression in murine tumor xenografts. The preclinical data corresponded remarkably well to first clinical PET/CT in cancer patients. ^68^ Ga-Trivehexin showed a high and persisting uptake in HNSCC (SUV_max_ = 13.1) as well as in primaries and liver metastases of PDAC (SUV_max_ about 10). No relevant uptakes were seen in other organs and tissues, except excretion-related in the kidneys and urinary tract which did not compromise tumor visualization in the investigated settings. We conclude that ^68^ Ga-Trivehexin possesses a high clinical value for PET imaging of all indications and conditions known to be frequently associated with elevated αvβ6-integrin expression, such as various carcinomas (pancreatic adeno, head-and-neck squamous, colorectal, cervical, lung adeno, and others), as well as fibrosis.

## Supplementary Information

Below is the link to the electronic supplementary material.

Supplementary file1 (PDF 1310 KB)

## Data Availability

The datasets used and/or analyzed during the current study are available from the corresponding author on reasonable request.
