# Supplementary material for: PET/CT imaging of head-and-neck and pancreatic cancer in humans by targeting the “Cancer Integrin” αvβ6 with Ga-68-Trivehexin
Source: Eur J Nucl Med Mol Imaging. 2021 Sep 24;49(4):1136–47. doi: 10.1007/s00259-021-05559-x (PMC8460406; doi:10.1007/s00259-021-05559-x)
Supplement: Supplementary file 1 — Supplementary file1 (PDF 1310 KB) [file 259_2021_5559_MOESM1_ESM.pdf]

Supporting Information

PET/CT Imaging of Head-and-Neck and Pancreatic Cancer in Humans by Targeting the "Cancer Integrin" αvβ6 with Ga-68-Trivehexin

Neil Gerard Quigley, Katja Steiger, Sebastian Hoberück, Norbert Czech, Maximilian Alexander Zierke, Susanne Kossatz, Marc Pretze, Frauke Richter, Wilko Weichert, Christian Pox, Jörg Kotzerke, Johannes Notni*

Synthesis of c[YRGDLAYp(*N*Me)K(pentynoic amide)] (Tyr2-alkyne)

General. The linear peptide sequence was assembled on 2-chlorotrityl resin (CC11006 by *Carbolution*, Germany), applying standard Fmoc strategy in 20 mL filter syringes with anhydrous dimethyl formamide (DMF) as solvent (except for resin loading, where dichloromethane (DCM) was used). For coupling, Fmoc-amino acids (2 eq) were dissolved in DMF (5 mL), pre-activated for 10 min with HATU (2 eq), HOBt (2 eq), and diisopropylethylamine (DIPEA, 6 eq), and gently shaken with the resin for a minimum of 3 h. Completeness of each coupling step was monitored by LC-MS after cleaving off a small sample of resin with a solution of 1,1,1,3,3,3-hexafluoropropane-2-ol (HFIP, 20% by volume) in DMF. Fmoc deprotection was done by treating the resin 2× for 10 min with DMF containing 20% (v/v) piperidine (6 mL); the resin was washed 4× with DMF before and after this step.

Resin loading and peptide assembly. 1 g resin was loaded with Fmoc-Gly (approx. loading capacity: 0.9 mmol), capped with a mixture of methanol (MeOH) and DIPEA (2:1 by volumes), and washed with DCM and DMF (4× each). Then, Fmoc-L-Arg(Pbf)-OH, Fmoc-L-Tyr(*t*Bu)-OH, and Fmoc-L-Lys(Dde)-OH were coupled with intermediate Fmoc deprotection.

N-methylation. After Fmoc removal from Lys, *N*-methylation was done via the Mitsunobu reaction. First, an *o*-NBS protection group was installed at the *N*-terminus by treating the resin with a solution of 2-nitrobenzenesulfonylchloride (*o*-NBS-Cl, 4 eq) and 2,4,6-collidine (10 eq) in DCM for 20 min. After washing with DCM (3×) and anhydrous tetrahydrofurane (THF; 5×), the resin was immersed in a solution of triphenylphosphine (PPh_3, 5 eq) in anhydrous MeOH, to which a solution of diisopropyl azodicarboxylate (DIAD, 5 eq) in a minimal amount of dry tetrahydrofurane (THF) was slowly added. After 15 min, the resin was washed with THF (5×) and DMF (5×), and the *o*-NBS protection group removed with 2-mercaptoethanol (10 eq) and 1,8-diazabicyclo[5.4.0]undec-7-ene (DBU) in DMF (6 mL) for 5 min.

Peptide assembly and cleavage. After washing with DMF (5×), coupling was continued with Fmoc-D-Pro-OH, Fmoc-L-Tyr(*t*Bu)-OH, Fmoc-L-Ala-OH, Fmoc-L-Leu-OH, and Fmoc-L-Asp(*t*Bu)-OH. The protected linear peptide was cleaved from the resin with a solution of HFIP (20 % by volume) in DCM (7 mL). Cleavage was repeated 2×, the resin washed with DCM (5×), all solutions combined and concentrated in vacuo.

Cyclization. To the crude linear peptide, DMF was added to reach a concentration of 1 mM, and vigorously stirred with anhydrous $NaHCO_3$ (5 eq) and diphenylphosphorylazide (DPPA, 3 eq) for 2 d at ambient temperature. The solution was concentrated in vacuo to 100 mL, filtered, and the Dde protecting group removed by addition of hydrazine hydrate (2 mL). Thereafter, the peptide intermediate was purified on a *Biotage* flash chromatography station on C18-RP cartridges, typically affording approx. 250 mg (≈ 20 % of theoretical yield).

Alkyne functionalization. A solution of 4-pentynoic acid (1.5 eq), HATU (1.5 eq), HOBt (1.5 eq), and DIPEA (2 eq) in a minimal amount of DMF was left to react for 15 min and added dropwise to a solution of the peptide intermediate (1 eq) and DIPEA (1 eq) in DMF. After 1 h at ambient temperature, the

solvent was evaporated and the residue treated with a mixture of trifluoroacetic acid / DCM / triisopropylsilane / H_2O (85:10:2.5:2.5 by volumes) for up to 3 h until LC-MS monitoring indicated complete deprotection. The deprotected peptide was precipitated into diethyl ether and purified by preparative HPLC, typically yielding > 100 mg of the title compound. MW (calcd. for $C_{56}H_{79}N_{13}O_{14}$): 1158.33. ESI-MS (positive mode): m/z = 1932.1 $[5M+3H^+]^{3+}$, 1739.0 $[3M+2H^+]^{2+}$, 1159.1 $[M+H^+]^+$, 580.4 $[M+2H^+]^{2+}$ (for MS spectra see Figure S2).

Synthesis of Trivehexin

Tyr2-alkyne (24.9 mg, 21.5 µmol, 3.3 eq) was added to a solution of TRAP(azide)$_3$ (5.4 mg, 6.5 µmol, 1 eq) and sodium ascorbate (65 mg, 326 µmol, 50 eq) in a minimal amount of H_2O. Cu^{II} acetate (1.56 mg, 7.84 µmol, 1.2 eq) was added and a brown precipitate immediately formed. Upon vortexing, the solution turned to a transparent green. The solution reacted for 1 h at 60 ˚C without stirring. After 1 h, Cu^{II} was removed from the TRAP chelator by reaction with a solution of 1,4,7-triazacyclononane-1,4,7-triacetic acid (NOTA) (39 mg, 130 µmol, 20 eq.) in water (1 mL), adjusted to pH 2.2 by addition of 1 M aq HCl, for 1 h at 60 ˚C. Purification by preparative RP-HPLC (gradient: 20–40% MeCN in H_2O, both containing 0.1% TFA, in 15 min) yielded 10.1 mg Trivehexin (36%). MW (calcd. for $C_{195}H_{291}N_{54}O_{51}P_3$): 4300.75. ESI-MS (positive mode): m/z = 1721.5 $[2M+5H^+]^{5+}$, 1434.7 $[M+3H^+]^{3+}$, 1076.2 $[M+4H^+]^{4+}$, 861.1 $[M+5H^+]^{5+}$, 717.8 $[M+6H^+]^{6+}$. The ^{nat}Ga-complex of Trivehexin for determination of affinities formed immediately upon mixing equal amounts (100 µL) of 1 mM aq. solutions of Trivehexin and $Ga(NO_3)_3$ hydrate. MW (calcd. for $C_{195}H_{288}N_{54}O_{51}P_3Ga$): 4367.44. ESI-MS (positive mode): m/z = 1747.9 $[2M+5H^+]^{5+}$, 1456.8 $[M+3H^+]^{3+}$, 1092.7 $[M+4H^+]^{4+}$, 874.4 $[M+5H^+]^{5+}$, 728.9 $[M+6H^+]^{6+}$ (for MS spectra see Figure S4).

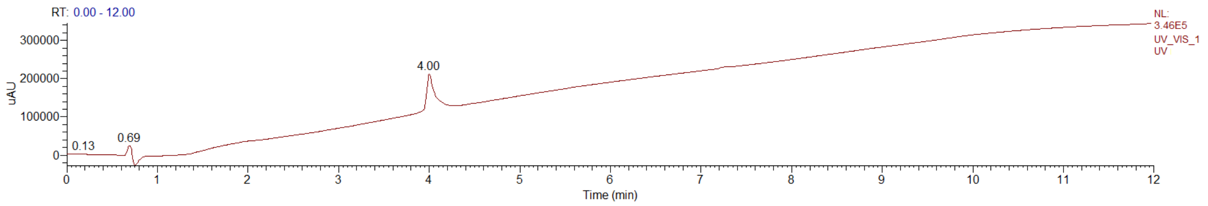

Figure S1: HPLC for [YRGDLAYp(*N*Me)K(pentynoic acid)] (Hypersil Gold aQ 175 Å, 3 µm, 150×2.1 mm analytical column; 0.7 mL/min; gradient: 5 to 95 % acetonitrile in water, both containing 0.1 % trifluoroacetic acid; detection: UV 220 nm).

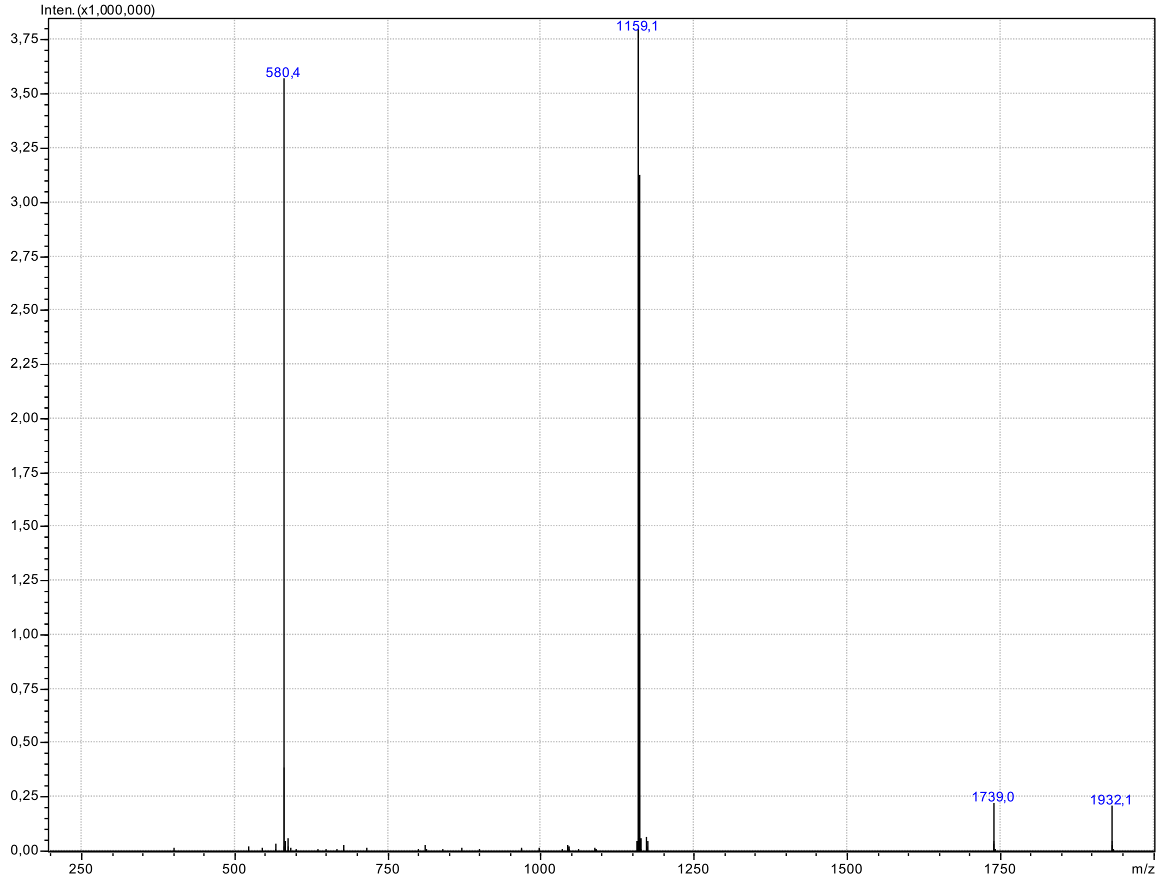

Figure S2: ESI-MS (positive mode) for [YRGDLAYp(*N*Me)K(pentynoic acid)]. *m/z* values correspond to molecular compositions as follows: 1932.1 $[5M+3H^+]^{3+}$, 1739.0 $[3M+2H^+]^{2+}$, 1159.1 $[M+H^+]^+$, 580.4 $[M+2H^+]^{2+}$.

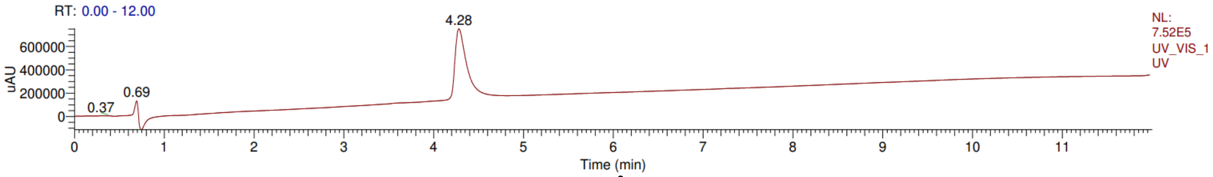

Figure S3: HPLC for Trivehexin (Hypersil Gold aQ 175 Å, 3 µm, 150×2.1 mm analytical column; 0.7 mL/min; gradient: 5 to 95 % acetonitrile in water, both containing 0.1 % trifluoroacetic acid; detection: UV 220 nm).

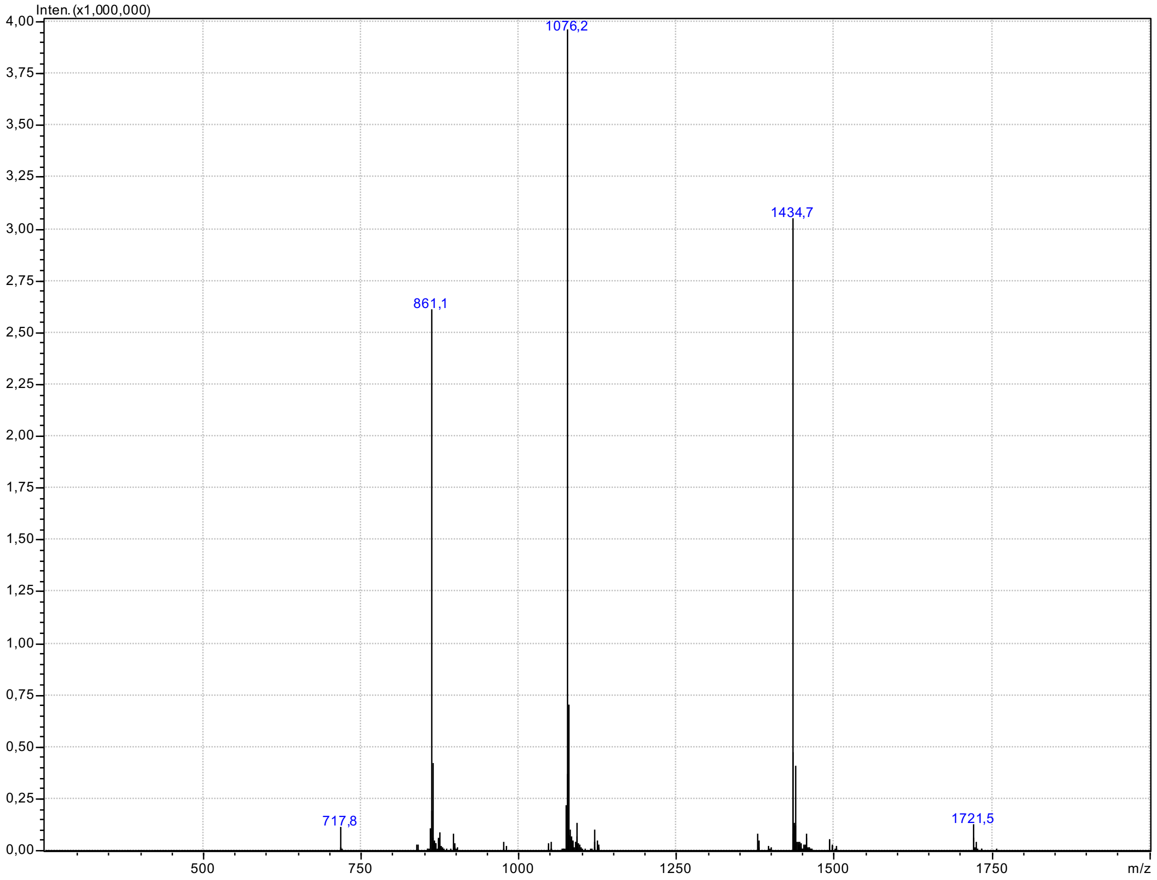

Figure S4: ESI-MS (positive mode) for Trivehexin. *m*/*z* values correspond to molecular compositions as follows: 1721.5 $[2M+5H^{+}]^{5+}$, 1434.7 $[M+3H^{+}]^{3+}$, 1076.2 $[M+4H^{+}]^{4+}$, 861.1 $[M+5H^{+}]^{5+}$, 717.8 $[M+6H^{+}]^{6+}$.

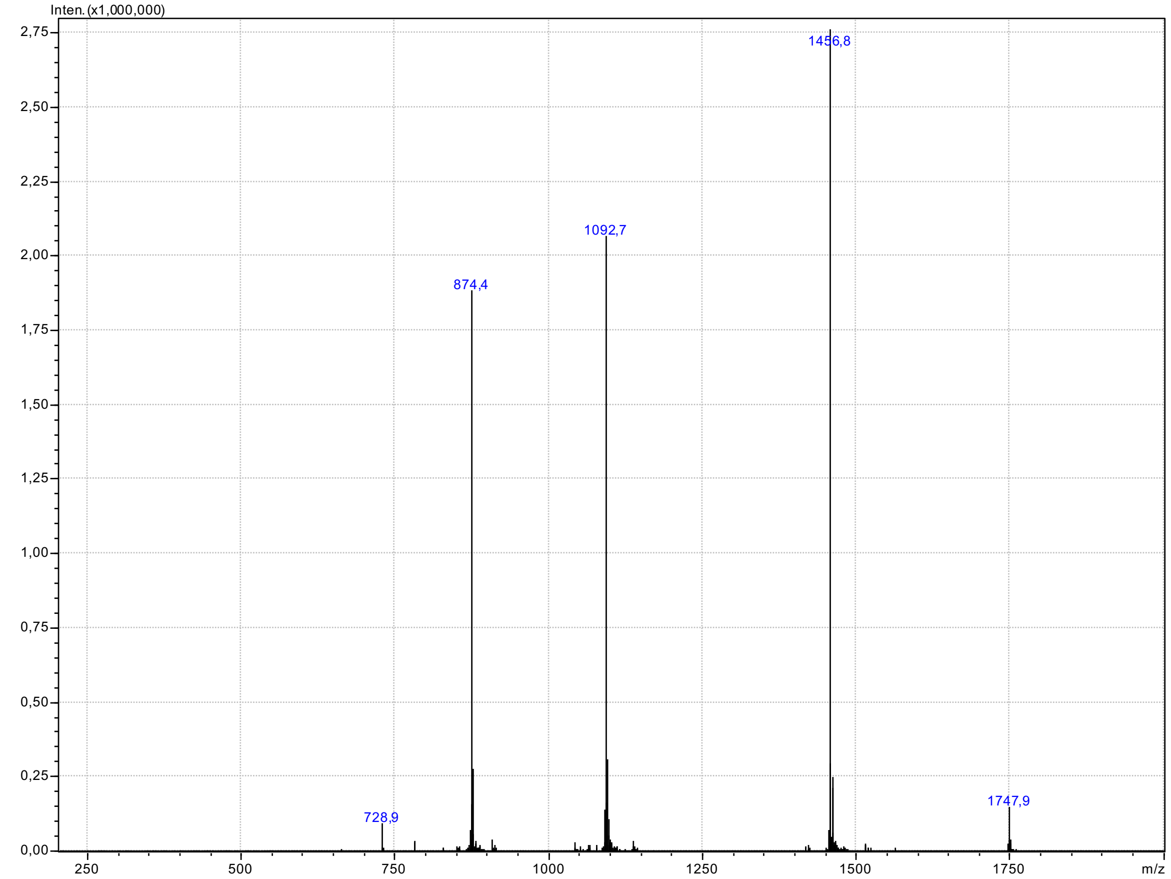

Figure S5: ESI-MS (positive mode) for ^{nat}Ga-Trivehexin. *m/z* values correspond to molecular compositions as follows: 1747.9 $[2M+5H^+]^{5+}$, 1456.8 $[M+3H^+]^{3+}$, 1092.7 $[M+4H^+]^{4+}$, 874.4 $[M+5H^+]^{5+}$, 728.9 $[M+6H^+]^{6+}$

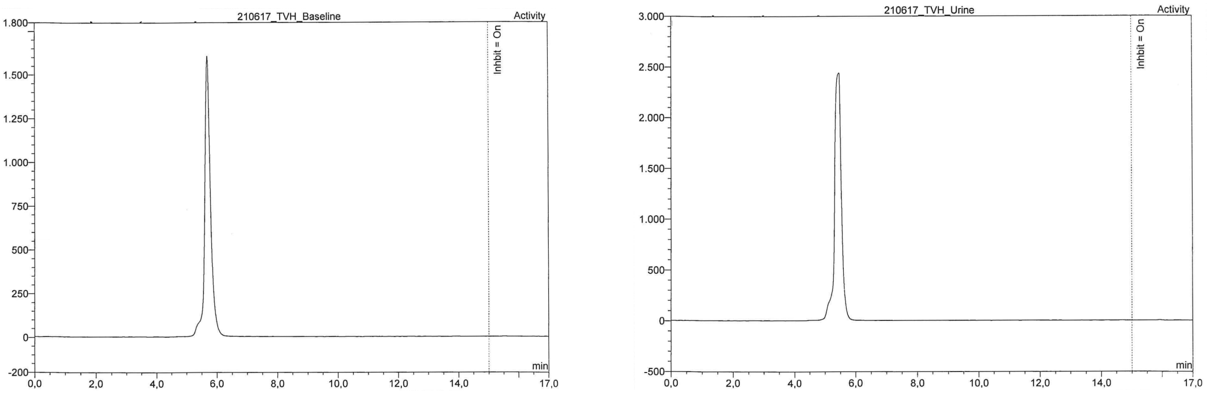

Figure S6: Radio-HPLC chromatograms for ^{68}Ga-Trivehexin injection solution in PBS (left) as well as mouse urine 30 min p.i. (right). Recorded on Shimadzu HPLC system; column: Chromolith performance 100×4.6 mm (Merck); flux: 3 mL / min, gradient: 5–60% MeCN in water, both containing 0.1 % trifluoroacetic acid (TFA), within 15 min.

Table S1: Biodistribution data (90 min p.i.) for ^{68}Ga-TRAP(AvB6)$_3$ without (n = 6; 66 ± 8 pmol) and with (n = 3) addition of 50 nmol of TRAP(AvB6)$_3$, in H2009 bearing SCID mice. Data are given as averages ± standard deviation. %IA/g = percent injected activity per gram tissue.

Organ/Tissue	^{68}Ga-TRAP(AvB6)$_3$		+ 50 nmol cold
	%IA/g	tumor/organ ratio	10 min prior to activity
Blood	4.01 ± 0.74	2.2 ± 0.5	2.39 ± 0.79
Heart (myocard)	2.13 ± 0.42	4.2 ± 0.8	1.93 ± 0.37
Lung	16.87 ± 5.34	0.6 ± 0.2	5.66 ± 1.47
Liver	7.55 ± 0.78	1.2 ± 0.2	7.65 ± 1.93
Spleen	5.21 ± 1.05	1.7 ± 0.4	2.56 ± 0.29
Pancreas	0.91 ± 0.22	10.1 ± 2.8	0.99 ± 0.15
Stomach (empty)	10.26 ± 1.62	0.9 ± 0.2	1.46 ± 0.22
Small intestine (empty)	4.20 ± 1.07	2.2 ± 0.4	1.16 ± 0.09
Large intestine (empty)	5.74 ± 1.36	1.6 ± 0.4	1.29 ± 0.16
Kidneys	90.74 ± 6.70	0.1 ± 0.0	61.56 ± 5.90
Adrenals	2.40 ± 0.38	3.7 ± 0.7	1.54 ± 0.43
Muscle	1.10 ± 0.22	8.1 ± 1.3	0.50 ± 0.11
Tumor H2009	8.73 ± 0.81		2.51 ± 0.24

Table S2: Biodistribution data (90 min p.i.) for ^{68}Ga-Trivehexin without (n = 5; 97 ± 13 pmol) and with (n = 3) addition of 50 nmol of Trivehexin, in H2009 bearing SCID mice. Data are given as averages ± standard deviation. %IA/g = percent injected activity per gram tissue.

Organ/Tissue	^{68}Ga-Trivehexin		+ 50 nmol cold
	%IA/g	tumor/organ ratio	10 min prior to activity
Blood	1.03 ± 0.21	7.5 ± 1.7	0.25 ± 0.15
Heart (myocard)	0.59 ± 0.11	13.2 ± 3.1	0.27 ± 0.03
Lung	4.47 ± 1.12	1.8 ± 0.6	0.98 ± 0.25
Liver	0.73 ± 0.11	10.5 ± 2.2	0.59 ± 0.08
Spleen	3.91 ± 0.91	2.0 ± 0.5	1.14 ± 0.13
Pancreas	0.27 ± 0.04	28.5 ± 4.2	0.10 ± 0.03
Stomach (empty)	6.24 ± 1.24	1.2 ± 0.2	0.37 ± 0.09
Small intestine (empty)	2.72 ± 1.15	3.1 ± 1.1	0.25 ± 0.06
Large intestine (empty)	4.36 ± 0.77	1.8 ± 0.5	0.35 ± 0.08
Kidneys	95.73 ± 12.59	0.1 ± 0.0	80.45 ± 17.63
Adrenals	0.50 ± 0.11	15.9 ± 4.3	0.37 ± 0.13
Muscle	0.68 ± 0.18	11.4 ± 2.6	0.07 ± 0.02
Tumor H2009	7.52 ± 1.21		0.69 ± 0.17

Table S3: Biodistribution data (90 min p.i.) for ^{68}Ga-Trivehexin (n = 5; 106 ± 35 pmol) in MDA-MB-231 bearing SCID mice. Data are given as averages ± standard deviation. %IA/g = percent injected activity per gram tissue.

Organ/Tissue	^{68}Ga-Trivehexin	
	%IA/g	tumor/organ ratio
Blood	0.72 ± 0.10	1.0 ± 0.2
Heart (myocard)	0.36 ± 0.04	2.1 ± 0.4
Lung	3.38 ± 0.20	0.2 ± 0.0
Liver	0.74 ± 0.07	1.0 ± 0.2
Spleen	2.30 ± 1.13	0.4 ± 0.2
Pancreas	0.26 ± 0.02	2.8 ± 0.5
Stomach (empty)	7.13 ± 1.61	0.1 ± 0.0
Small intestine (empty)	1.79 ± 0.38	0.4 ± 0.1
Large intestine (empty)	2.47 ± 0.49	0.3 ± 0.1
Kidneys	70.59 ± 20.00	0.0 ± 0.0
Adrenals	0.54 ± 0.44	4.3 ± 6.7
Muscle	0.36 ± 0.10	2.2 ± 1.0
Tumor MDA-MB231	0.72 ± 0.13	

Table S4: Standard uptake values (SUV_{mean}; SUV_{max} in parentheses) for selected areas of a ^{68}Ga-Trivehexin PET scan (172 MBq, total peptide amount 5 nmol) (Figure 3)

Organ	13 min p.i.	44 min p.i.	97 min p.i.
Blood (thoracic aorta)	1.9 (2.9)	1.4 (2.3)	1.5 (2.8)
Heart	2.1 (3.8)	1.7 (3.1)	2.0 (3.6)
Lung (avg.)	0.78 (1.5)	0.94 (1.8)	1.1 (2.1)
Liver	2.6 (4.9)	2.7 (5.1)	2.9 (5.5)
Spleen	2.3 (3.4)	2.2 (4.0)	2.5 (4.8)
Pancreas	3.4 (5.9)	2.4 (4.2)	2.6 (4.5)
Stomach	9.4 (16)	7.5 (14)	5.4 (9.8)
Small intestine	2.9 (5.0)	2.5 (4.1)	3.6 (6.5)
Large intestine (ascending)	2.0 (3.4)	1.5 (2.4)	2.0 (3.6)
Kidneys (avg.)	34 (52)	36 (59)	44 (73)
Urinary bladder	24 (41)	44 (82)	55 (84)
Adrenals (avg.)	1.9 (2.8)	2.8 (3.1)	1.9 (2.9)
Muscle	1.6 (2.8)	1.7 (3.1)	1.8 (3.3)
Eye (avg.)	3.3 (5.9)	3.1 (5.2)	2.3 (4.0)
Coeliac plexus (avg.)	2.3 (3.6)	2.3 (3.9)	2.2 (3.8)
Choroid plexus	1.6 (2.6)	1.4 (2.3)	2.0 (3.4)

Table S5: Dose estimates for a bolus i.v. injection of 175 MBq ^{68}Ga-Trivehexin, calculated with OLINDA V1.1 based on the organ residence times calculated from data shown in **Table S4**.

Organ	Dose (mGy/MBq)
Adrenals	2.81E-02
Brain	6.78E-03
Breasts	7.23E-03
Gallbladder Wall	1.43E-02
LLI Wall	2.81E-02
Small Intestine	5.69E-02
Stomach Wall	4.25E-02
ULI Wall	2.24E-02
Heart Wall	1.91E-02
Kidneys	4.77E-01
Liver	3.73E-02
Lungs	1.59E-02
Muscle	8.76E-03
Ovaries	1.18E-02
Pancreas	3.41E-02
Red Marrow	8.38E-03
Osteogenic Cells	1.14E-02
Skin	7.07E-03
Spleen	3.39E-02
Testes	7.92E-03
Thymus	8.11E-03
Thyroid	7.45E-03
Urinary Bladder Wall	9.29E-02
Uterus	1.25E-02
**Total Body**	**1.24E-02**
